# Aktueller Stand der Impfung gegen SARS-CoV-2

**DOI:** 10.1007/s00393-021-00966-9

**Published:** 2021-02-02

**Authors:** Christof Iking-Konert, Christof Specker, Klaus Krüger, Hendrik Schulze-Koops, Peer Aries

**Affiliations:** 1 III. Medizinische Klinik und Poliklinik Sektion Rheumatologie, Universitätsklinikum Hamburg-Eppendorf, Martinistr. 52, 20251 Hamburg, Deutschland; 2 Klinik für Rheumatologie und Klinische Immunologie, Evangelisches Krankenhaus Kliniken Essen-Mitte, Essen, Deutschland; 3 Rheumatologisches Praxiszentrum, München, Deutschland; 4 Sektion Rheumatologie und Klinische Immunologie, Medizinische Klinik und Poliklinik IV, Ludwig-Maximilians-Universität München, München, Deutschland; 5 Rheumatologie im Struenseehaus, Hamburg, Deutschland

**Keywords:** SARS-CoV-2, COVID-19, Impfung, STIKO Empfehlung, mRNA Vakzine, SARS-CoV-2, COVID-19, Vaccination, STIKO recommandation, mRNA vaccination

## Abstract

Am 21.12.2020 hat die EMA mit der mRNA-Vakzine BNT162b2 der Firmen BioNTech/Pfizer (Comirnaty®, BioNTech, Mainz, Deutschland; Pfizer, New York City, NY, USA) den ersten Impfstoff gegen SARS-CoV‑2 in der Europäischen Union zugelassen, am 06.01.2021 folgte bereits der zweite (Moderna®, Moderna, Cambridge, MA, USA). Viele weitere Zulassungen werden in Kürze folgen. Mit der Verfügbarkeit der Impfung sind bei Patienten/-innen genau wie bei Ärzten/-innen viele Fragen aufgekommen, die in diesem Hot-Topic-Artikel so aktuell wie möglich adressiert werden sollen.

## Abstract (en)

On 21 December 2020 the European Medicines Agency (EMA) approved the mRNA vaccine BNT162b2 (Comirnaty®, BioNTech, Mainz, Germany; Pfizer, New York City, NY, USA) as the first vaccine against SARS-CoV‑2 in the European Union and the second vaccine (Moderna®, Moderna, Cambridge, MA, USA) followed on 6 January 2021. Many more approvals will follow within a short period of time. With the availability of the vaccination many questions have arisen for patients as well as for physicians, which are addressed as up to date as possible in this hot topic article.

Am 21.12.2020 hat die European Medicines Agency (EMA) den ersten Impfstoff gegen SARS-CoV‑2 in der Europäischen Union (EU) zugelassen [1], am 27.12.2020 wurden in Deutschland bereits die ersten Personen geimpft. Es handelt sich um die mRNA-Vakzine BNT162b2 der Firmen BioNTech/Pfizer (Comirnaty®, BioNTech, Mainz, Deutschland; Pfizer, New York City, NY, USA). Die Zulassung wurde für Personen ab 16 Jahre erteilt, für Kinder und Jugendliche unter 16 Jahren ist der Impfstoff zunächst noch nicht zugelassen. Der Impfstoff wurde zuvor bereits neben der mRNA-1273-Vakzine der Firma Moderna (COVID-19-Impfstoff Moderna®, Cambridge, MA, USA) in den USA durch die FDA sowie in anderen Ländern wie Kanada, Großbritannien oder der Schweiz in einem Eilverfahren zugelassen. In der EU erfolgte die Zulassung für den Moderna-Impfstoff noch am 06.01.2021, für Erwachsene ab dem 18. Lebensjahr.

Beide Impfstoffe zeigen in Studien eine nahezu identisch gute Wirksamkeit und Verträglichkeit. In der Phase-III-Studie an mehr als 20.000 Probanden im Verumarm des Impfstoffes BNT162b2 wird z. B. eine Impfeffektivität von 95 % angegeben, die auch in der kritischen Altersgruppe über 65 Jahre erreicht werden konnte (Tab. 2). Auch der sekundäre Endpunkt „Vermeidung von schweren COVID-19-Infektionen“ wurde erfüllt. Es fanden sich keine Wirksamkeitsunterschiede in Subgruppenanalysen (u. a. hinsichtlich Geschlecht, ethnischer Herkunft oder bestehender Risikofaktoren).

Die häufigsten berichteten Nebenwirkungen (NW) waren lokale Reaktionen (83 % vs. 14 %), aber auch systemische NW wie Fatigue (47 % vs. 33 %) oder Myalgien (21 % vs. 11 %) wurden beobachtet. Schwere oder lebensbedrohliche NW wurden unter dem Impfstoff mit 0,3 % statistisch nicht häufiger angegeben als unter Placebo mit 0,1 %. Bei beiden Impfstoffen wurden wenige, transiente Fazialisparesen beobachtet, die mit der Impfung in Zusammenhang stehen könnten. Wie bei allen Medikamenten können sehr seltene Ereignisse durch Zulassungsstudien noch nicht ausgeschlossen werden.

Aktuell gehen sowohl der Hersteller als auch die Ständige Impfkommission (STIKO) und das Robert-Koch-Institut (RKI) davon aus, dass die Impfstoffe auch gegen die mutierte und hoch kontagiöse Varianten von SARS-CoV‑2, u. a. VOC 202012/01 („variant of concern“, vorher VUI: „variant under investigation“), wirksam sein werden.

Zulassungen weiterer Impfstoffe gegen SARS-CoV‑2 werden auch in der EU in Kürze erwartet, mit Stand Dezember 2020 wird weltweit an mehr als 230 Impfstoffkandidaten geforscht. Eine Übersicht über die 2 in der EU am 15.1.21 zugelassenen Vakzine geben Tab. 1 und 2.

**Table 1:** 

Firma	Name der Vakzine	Typ der Vakzine	Impfschema	Zulassung
BioNTech/Pfizer	BNT162b2 (Comirnaty®)	mRNA Spike Protein	Tag 0 und 21 i.m.^a^	Zulassung durch EMA am 21.12.2020
Moderna	mRNA-1273 (Covid-19-Vaccine Moderna, kurz Moderna ®)	mRNA Spike-Protein	Tag 0 und 28 i.m.^a^	Zulassung durch EMA am 06.01.2021

*SARS-CoV-2* severe acute respiratory syndrome coronavirus 2, *mRNA-1273*  messenger ribonucleic acid

^a^Eine begonnene Impfserie muss mit demselben Impfstoff beendet werden. Die 2. Gabe sollte spätestens innerhalb des durch die Studien abgedeckten Zeitraums (42 Tage) erfolgen

**Table 2:** 

Vakzine	Relevante Studien Charakteristika	Wirksamkeit	Nebenwirkungen (Auszüge)
BNT162b2	Phase-II/III-Studie 21.720 Probanden > 16a mit mind. 1 Dosis; 20.519 mit 2 Dosen 21.728 Probanden mit Placebo Analyse für den 1. Endpunkt: 18.242 Verum und 18.379 Placebo Follow-up 2,214 Proband Jahre im Verumarm	COVID-19-Fälle gesamt im Verumarm: 8 COVID-19-Fälle gesamt im Placeboarm: 162 Risikoreduktion: 95 % COVID-19-Fälle >65a im Verumarm (*n* = 3848): 1 COVID-19-Fälle >65a im Placeboarm (*n* = 3880): 19 Risikoreduktion: 94,7 % COVID-19-Fälle >75a im Verumarm (*n* = 774): 0 COVID-19-Fälle >75a im Placeboarm (*n* = 785): 5 Risikoreduktion: 100 %	Sehr häufig > 1/10 Arthralgien, Myalgien, Kopfschmerzen, Fatigue, Fieber, lokale Injektionsreaktionen Häufig > 1/100 < 1/10 Übelkeit, lokale Reaktionen Ungewöhnlich > 1/1000 < 1/100 Schmerzen der Extremitäten, Lymphadenopathie, Schlafstörungen Selten oder unbekannt > 1/10.000 < 1/1000 Akute Fazialisparese, Anaphylaxie
mRNA-1273	Studie 301: i.m. 100 μg mRNA-1273-Vakzine (*n* = 15,181) oder Placebo (*n* = 15,170) > 18a	COVID-19-Fälle gesamt im Verumarm: 5 COVID-19-Fälle gesamt im Placeboarm: 15 Risikoreduktion: 94,5 % COVID-19-Fälle >65a im Verumarm: 0 COVID-19-Fälle >65a im Placeboarm: 19 Risikoreduktion: 100 %	Häufig: Lokalreaktionen (mild – moderat) mit Schmerz (84 % vs. 20 %) Systemisch: Müdigkeit (68,5 %), Kopfschmerz (63,0 %), Myalgien (59,6 %), Arthralgien (44,8 %) Lymphadenopathie (Verum 1,1 % vs. 0,63 % Placebo) Hypersensitivität: 1,5 % Impfgruppe vs. 1,1 % Placebo (keine Anaphylaxie) 13 Todesfälle (6 Verum, 7 Placebo) „Serious AE“: (1 % in der mRNA-1273-Gruppe vs. 1 % in der Placebogruppe)

*mRNA-1273* messenger ribonucleic acid, *COVID-19* coronavirus disease 2019, *AE* adverse event a Jahre

Grundsätzlich sollte jeder, der sich gegen SARS-CoV‑2 impfen lassen möchte, eine Impfung bekommen (Individualschutz). Eine sog. Herdenimmunität (oder Gemeinschaftsschutz) wird nach mathematischen Modellen bei einer Impfquote bzw. Immunität von ca. 70 % in der Bevölkerung erreicht, abhängig natürlich auch von der Immunität in einer Population nach durchgemachten Infektionen (gesicherte COVID-19-Fälle in Deutschland bis Anfang Januar mehr als 1,7 Mio.). Abhängig davon, wie viel Impfstoff in Deutschland zur Verfügung steht und wie die Verteilung und Verabreichung des Impfstoffs organisiert wird, wird das Ziel dieser Herdenimmunität früher oder später erreicht werden. Auch die Akzeptanz der Impfung in der Bevölkerung wird entscheidend für den Erfolg der Impfstrategie sein. In Deutschland kam es zu Beginn der Impfkampagne zu erheblichen Problemen und Verzögerungen.

Es handelt sich bei den bisher zugelassenen SARS-CoV‑2.Vakzinen um sog. Messenger-RNA(mRNA)-Impfstoffe. Hierbei werden nicht Virusproteine oder nicht mehr vermehrungsfähige Vektorviren geimpft, sondern die biologische Information (mRNA) für die Synthese von virusidentischen Proteinen im Organismus des Geimpften. Diese mRNA wird nach der Proteinsynthese abgebaut und gelangt nicht in das Erbgut, es handelt sich also nicht um eine „Gentherapie“. Ursprünglich wurde die Technik als Immuntherapie gegen diverse Tumoren entwickelt, wofür sie aber noch nicht zur zugelassenen Anwendung kommt.

Noch nie hat eine Impfstoffentwicklung (oder auch eine andere Medikamentenentwicklung) dermaßen schnell zu einer Zulassung geführt, wie es bei den Impfstoffen gegen SARS-CoV‑2 der Fall war. Dies ist durch einen massiven „medical-need“ aufgrund der weltweiten, disruptiven Auswirkungen der Corona-Pandemie und die konzertierten Bemühungen von Wissenschaft und Politik zu erklären, die Entwicklung rasch voranzubringen, ohne auf wesentliche Aspekte der Sicherheit des Impfstoffes zu verzichten. Außerdem konnten die Firmen auf bereits vorliegende Erfahrungen im Rahmen der Entwicklung der mRNA-Vakzine gegen Tumoren zurückgreifen. mRNA-Impfstoffe sind darüber hinaus schnell und in großen Mengen herstellbar. Neben diesen wissenschaftlichen Anstrengungen haben die parallele Durchführung der Prüfphasen und eine kontinuierliche Einreichung der Studienergebnisse bei den Zulassungsbehörden die notwendige Entwicklungszeit verkürzt. Bei Kritikern führt dies zu unbegründeter, aber leider häufig lauter und emotional vorgetragener Sorge und Skepsis („Geschwindigkeit vor Sorgfalt“). In einer der führenden wissenschaftlichen Fachzeitschriften (*Science*) wurde die Entwicklung von Impfstoffen gegen SARS-CoV‑2 in bislang nicht erreichter Geschwindigkeit zum „Breakthrough of the Year“ erklärt [2]. Auch muss betont werden, dass im beschleunigten Bewertungsverfahren („accelerated assessment“) die regulatorische Bewertungszeit zwar verkürzt ist, die Sicherheitsstandards sich jedoch in keiner Weise von einer ordentlichen Zulassung unterscheiden. Nach Umfragen des RKI (www.corona-monitor.de) gaben zuletzt (Stand 15.12.2020) trotzdem nur noch 49 % der Befragten an, sich „eher impfen zu lassen“, nachdem dies im Frühjahr noch 79 % der Befragten wollten. Beunruhigend war auch, dass in dieser Befragung die Bereitschaft bei medizinischem Personal eher noch geringer war als in der Allgemeinbevölkerung. Erste Erfahrungen während der jetzt begonnenen Impfkampagne in Alten- und Pflegeheimen und Kliniken bestätigen diesen Trend. Eine generelle Impfpflicht soll es vorerst nicht geben, über eine Impfpflicht bestimmter Zielgruppen wird aber bereits diskutiert. Die Impfung wird für die gesamte Bevölkerung kostenlos sein.

Die Ad-hoc-Kommission COVID-19 und der Vorstand der Deutschen Gesellschaft für Rheumatologie e. V. (DGRh) haben bereits im Dezember 2020 zusammen mit der Kommission Pharmakotherapie der DGRh zum Thema „Impfung gegen SARS-CoV-2“ eine erste Stellungnahme verfasst und auf der Internetseite der Fachgesellschaft publiziert [3]. Diese frühen Empfehlungen für Patienten und Therapeuten sollten drängende Fragen zu dem Thema beantworten und Sorge und Ängste nehmen. Die Akzeptanz der neuen Impfung soll so bei Patienten, aber auch bei Kolleginnen und Kollegen, erhöht werden.

Aktuell gibt es noch keine Daten zur Sicherheit und Effektivität der verschiedenen SARS-CoV-2-Vakzine bei Patienten mit entzündlich rheumatischen Erkrankungen bzw. unter immunsuppressiver/immunmodulierender Therapie. Entsprechend den Protokollen waren sowohl bei Moderna als auch bei BioNtech/Pfizer Patienten mit (bekannten) Autoimmunerkrankungen von einer Studienteilnahme ausgeschlossen. Für eine Impfung gegen SARS-CoV‑2 gilt wie für sämtliche Impfungen, die bislang bei Patienten mit Autoimmunerkrankungen und insbesondere unter immunsuppressiver/immunmodulierender Therapie regelmäßig zum Einsatz kommen, dass alle bislang bekannten Totimpfstoffe (Vakzine auf der Grundlage nicht-replizierbarer Vektoren, Vakzine auf der Grundlage adjuvantierter Proteine) bei diesen Patientengruppen gefahrlos einsetzbar sind. Lebendimpfstoffe (Vakzine auf der Grundlage attenuierter Viren) sollen hingegen bei Patienten mit supprimiertem Immunsystem generell mit ganz wenigen, im Einzelfall zu begründenden Ausnahmen nicht eingesetzt werden. Wichtig für die Bewertung der Impfstoffe gegen SARS-CoV‑2 ist die Tatsache, dass alle bislang entwickelten und derzeit in fortgeschrittenem Stadium der Testung befindlichen SARS-CoV-2-Vakzine Totimpfstoffe sind. Zu diesen zählen auch die mRNA-Vakzine.

Der Einsatz einer mRNA-Vakzine kann nach aktuellem Kenntnisstand bei Patienten mit entzündlich rheumatischen Erkrankungen und bei Patienten unter immunsuppressiver/immunmodulierender Therapie daher empfohlen werden. Die aktuellen Meldungen nach Zulassung über (teils schwere) allergische Reaktionen – mutmaßlich gegen Inhaltstoffe wie PEG – sind keine Besonderheit eines mRNA-Impfstoffes und geben, insbesondere auch unter Nutzen-Risiko-Abwägung, keinen Anlass, von dieser Empfehlung abzuweichen. Dem Paul-Ehrlich-Institut (PEI) wurden im Zeitraum vom 27.12.2020 bis 10.01.2021 bisher insgesamt 325 Nebenwirkungsverdachtsfälle gemeldet. In 51 Fällen wurde über schwerwiegende Reaktionen berichtet, das entspricht einer Melderate für schwerwiegende Nebenwirkungen von 0,08 pro 1000 Impfungen. Sieben Todesfälle im Alter von 79 bis 93 Jahre wurden berichtet, die nicht sicher im Zusammenhang mit der Impfung standen [4] Relevante unerwünschte Nebenwirkungen müssen weiter konsequent gemeldet werden [5]. Im Falle einer allergischen Reaktion darf keine zweite Impfung erfolgen. Eine Aufklärung über potenzielle Nebenwirkungen soll vor der Impfserie erfolgen.

Aus grundsätzlichen Überlegungen zur Effektivität einer Impfung sollte die Immunsuppression zum Zeitpunkt der Impfung so gering wie möglich sein. Das Risiko einer Reaktivierung der rheumatologischen Erkrankung nach Absetzen einer immunmodulierenden/immunsuppressiven Therapie zur potenziellen Verbesserung der Impfantwort wird aber als so erheblich eingeschätzt, dass nicht empfohlen wird, die bestehende Therapie wegen einer Impfung zu pausieren. Als Ausnahme gilt die Gabe von B‑Zell-depletierenden Substanzen (v. a. Rituximab). Hier sollte unter Abwägung der Gefahr einer Reaktivierung der Grunderkrankung einerseits und der Verbesserung einer Impfantwort andererseits eine Streckung des Therapieintervalls oder die Umstellung auf alternative Therapien erwogen werden. Als bester Zeitpunkt einer Impfung wird wie bei anderen Impfungen auch ein Zeitraum von ca. 4 Wochen vor der (Re‑)Therapie angenommen.

Für die Umsetzung der Impfempfehlungen werden die Bundesländer bzw. deren Impfstellen zuständig sein. Die Abläufe in den einzelnen Ländern und Städten sind aktuell trotz intensiver Bemühungen weiter stockend und regional sehr unterschiedlich effektiv. Impfberechtigte, die allein aufgrund des Alters zu einer der Priorisierungsgruppen zu zählen sind, erhalten in den meisten Bundesländern eine schriftliche Einladung zur Impfung. Wie und wann die anderen Personen geimpft werden können bzw. einen Termin vereinbaren können, ist regional unterschiedlich geregelt. Ob man bereits impfberechtigt ist und wann man einen Termin bekommt, können Patienten aber z. B. in 4 Bundesländern schon hier prüfen: https://www.impfterminservice.de/impftermine.

Rheumatologen/-innen sollten sich z. B. über die Seiten der Gesundheitsämter oder KVen über Details informieren. Sowohl die STIKO als auch verschiedene weitere Schwerpunktgesellschaften neben der DGRh (Übersicht auf der Seite der DGIM) haben Stellungnahmen zur Dringlichkeit der Impfung von bestimmten Patientengruppen veröffentlicht oder stellen sie zurzeit zusammen. Patienten sollten unter Berücksichtigung der offiziellen zum jeweiligen Zeitpunkt gültigen Stellungnahmen der STIKO Atteste für eine Priorisierung erhalten, damit sie die Zugehörigkeit zu den von der STIKO vorgegebenen Priorisierungsgruppen nachweisen können (s. unten).

Aktuell kann keine evidenzbasierte Empfehlung zu einer Impfung nach erfolgreich überstandener COVID-19-Erkrankung ausgesprochen werden. Nach Expertenmeinung sollten diese Personengruppen vorerst nicht geimpft werden, da von einer natürlichen Immunität gegen das Virus ausgegangen wird. Am 08.01.2021 hat jedoch der Bundesgesundheitsminister unter dem Eindruck der nach einer Infektion zeitlich begrenzten Präsenz neutralisierender Antikörper gegen SARS-CoV-2 öffentlich die Empfehlung ausgesprochen, dass sich auch ehemals Infizierte impfen lassen sollten. Eine Testung auf stattgehabte COVID-19 vor der Impfung wird nicht empfohlen. Ein Risiko durch eine Impfung bei durchgemachter Infektion ist nicht bekannt. Personen, die zwischen der 1. und 2. Impfung positiv auf SARS-CoV‑2 getestet werden, sollten nicht zum zweiten Mal geimpft werden. Andere planbare Impfungen sollten mit einem Mindestabstand von 14 Tagen zu der SARS-CoV-2-Serie gegeben werden. Die Impfempfehlung gilt aktuell für Jugendliche über 16 Jahre (BionTech/Pfizer) und Erwachsene, bzw. ab 18 Jahren (Moderna). Zur Impfung in der Schwangerschaft und Stillzeit liegen aktuell noch keine Daten vor, eine generelle Impfung empfiehlt die STIKO aktuell noch nicht. Schwangeren mit hohem Risiko kann nach strenger Abwägung von Nutzen und Risiko sowie Aufklärung eine Impfung angeboten werden. Da die Daten zur Wirksamkeit einer Transmission des Virus nach einer Impfung noch uneinheitlich sind, wird auch nach Impfung noch die strenge Einhaltung der allgemeinen Schutz- und Hygienemaßnahmen empfohlen.

## Impfpriorisierung

Die Verfügbarkeit der Impfstoffe ist weiter massiv eingeschränkt. Das Bundesgesundheitsministerium (BGM) rechnet im 1. Quartal 2021 mit ca. 11–13 Mio. zur Verfügung stehenden Impfdosen, was für maximal 6,5 Mio. Personen reichen würde. Logistische Hürden behindern das sehr zeitnahe Durchimpfen der gesamten Bevölkerung ebenfalls. Zu Beginn wird der Impfstoff deshalb zunächst nur Menschen mit einem besonderen Infektionsrisiko (z. B. Personen in höherem Lebensalter oder medizinisches Personal in der Betreuung von COVID-19-Erkrankten), Personen, die ein hohes Risiko im Falle einer Infektion haben (wie Transplantierte oder Patienten mit schwerem Immundefekt) und damit ein Risiko für einen schweren oder tödlichen Verlauf (s. unten), angeboten werden, um die Verbreitung von SARS-COV‑2 möglichst effektiv zu verhindern und besonders vulnerable Gruppen zu schützen.

Die COVID-19-Arbeitsgruppe der STIKO wurde deshalb mit der Entwicklung von Impfstrategien beauftragt. Als Leitfaden für diese Impfempfehlung diente ein gemeinsames Positionspapier der STIKO, Leopoldina und des Deutschen Ethikrats [6].

Der endgültige Beschluss der STIKO für die Empfehlung der COVID-19-Impfung und die dazugehörige wissenschaftliche Begründung wurden kürzlich publiziert [7] und haben am 08.01.2021 bereits eine Aktualisierung erfahren [8]. Darin wird ein stufenweises Vorgehen bei der Impfung gegen COVID-19 empfohlen und in 6 Priorisierungsstufen eingeteilt. Diese Priorisierung wird so lange empfohlen, bis genügend Impfstoff zur Verfügung steht, um allen Personen eine Impfung anbieten zu können.

Vor der Veröffentlichung der STIKO wurden diverse Fachgesellschaften, u. a. die DGRh, zu einer Stellungnahme zu dem Entwurf geladen. In einer Sitzung der Schwerpunktgesellschaften am 02.12.2020 wurde entschieden, ein gemeinsames Communiqué zu dem Thema „Definition von Risikogruppen für schwere oder komplikative COVID-19-Verläufe“ seitens der DGIM zu erstellen. Es sollten alle Patienten der Inneren Medizin bewertet werden, um so Partikularinteressen einzelner Patientengruppen oder Fachgesellschaften zu vermeiden. Eine Bevorzugung einzelner Gruppen wäre nach Meinung der DGIM unethisch und medizinisch falsch. Dieser Meinung hat sich die DGRh in ihrer an die DGIM und die STIKO Ende Dezember 2020 weitergeleiteten Stellungnahme ohne Einschränkung angeschlossen.

Der größte Risikofaktor für schwere Verläufe war in den Analysen der STIKO eindeutig das Alter der Patienten mit einer OR von > 4,5 für die Gruppe der über 70-Jährigen. Wie sich die Analyse bezüglich Hospitalisierung und Mortalität einzelner Erkrankungen auswirken würde, ist beispielhaft in Tab. 3 für Patienten mit entzündlich rheumatischen Erkrankungen und für 2 wesentliche Komorbiditäten aufgezeigt, wobei jeder Wert < 2 von der Kommission als „moderates“ Risiko eingeschätzt wurde. Entsprechend den Ergebnissen stufte die STIKO Patienten mit rheumatologischen Erkrankungen oder unter Immunsuppression in Stufe 3 und/oder 4 ein (s. Tab. 4 und 5).

**Table 3:** 

Erkrankung/Gruppe	Odds Ratio (95 %-KI) für Hospitalisierung *n* Studien/*n* Patienten	Odds Ratio (95 %-KI) für Mortalität *n* Studien/*n* Patienten	Evidenz Qualität nach STIKO
Autoimmunerkrankung	1,08 (1,01–1,17) 1/109.367	1,19 (1,06–1,33) 1/8582	+++
Immunkompromittierung	1,14 (0,75–1,75) 1/1542	1,39 (1,13–1,7) 1/2490	+++
Rheumatische Erkrankungen	1,4 (1,1–1,8) 1/9519	0,87 (0,66–1,16) 1/2490	+++
Diabetes mellitus	2,09 (1,83–2,38) 6/17.303	1,37 (1,22–1,56) 9/8388	++++
Chronische Nierenerkrankung	2,24 (1,28–3,9) 4/15.809	1,7 (1,46–1,97) 8/8296	+++

**Table 4:** 

	Impfverordnung des BMG Priorität: erhöht (Stufe 3 von 3)		
	STIKO Priorität: Stufe 3 (3 von 6)		STIKO Priorität: Stufe 4 (4 von 6)
	DGRh Priorität: Stufe 2	DGRh Priorität: Stufe 3	DGRh-Priorität: Stufe 4
Erkrankung	Systemische Verläufe von entzündlich rheumatischen Erkrankungen (wie Vaskulitiden und Kollagenosen) mit aktiver Organbeteiligung von Lunge, Herz, Niere, ZNS bzw. mit fortgeschrittener Organschädigung Primäre und sekundäre Immundefizienz – IgG-Spiegel < 400 mg/dl – Leuko- und/oder Lymphopenie WHO Grad III–IV	Systemische Verläufe von entzündlich rheumatischen Erkrankungen in Remission (wie Vaskulitiden und Kollagenosen) ohne aktive Organbeteiligung bzw. ohne relevante Organschädigung	Entzündlich rheumatische Erkrankungen wie rheumatoide Arthritis, Psoriasisarthritis oder Spondyloarthritis ohne Anhalt für Organbeteiligung
Therapie	Absehbarer mittel- bis langfristiger Bedarf von systemischen Steroiden > 10 mg Prednisolonäquivalent/Tag Therapie mit Rituximab^a^ und Cyclophosphamid	Absehbarer mittel- bis langfristiger Bedarf von systemischen Steroiden 5–10 mg Prednisolonäquivalent/Tag	Kurzfristige Gabe von Steroiden und/oder Low-dose-Dauertherapie Prednisolon-äquivalent/Tag < 5 mg – bDMARD (Ausnahme Rituximab) – tsDMARD – csDMARD

*bDMARD* biologische „disease modifying drugs“, *tsDMARD* „target synthetic disease modifying drugs“, *csDMARD* „conventional synthetic disease modifying drugs“, *ZNS* Zentralnervensystem, *IgG* Immunglobulin G, *WHO* World Health Organization,

^a^Wenn möglich Impfung vor dem Start der Therapie oder am Ende des Therapieintervalls, üblicherweise 4 Wochen vor der nächsten RTX-Therapie

**Table 5:** 

Priorität	Anzahl geschätzt
**Höchste Priorität** Über 80-Jährige Personen, die in stationären Einrichtungen für ältere oder pflegebedürftige Menschen behandelt, betreut oder gepflegt werden oder tätig sind Pflegekräfte in ambulanten Pflegediensten *Beschäftigte in medizinischen Einrichtungen mit hohem Expositionsrisiko wie Intensivstationen, Notaufnahmen, Rettungsdienste, als Leistungserbringer der spezialisierten ambulanten Palliativversorgung, SARS-CoV-2-Impfzentren und in Bereichen mit infektionsrelevanten Tätigkeiten* *Beschäftigte in medizinischen Einrichtungen, die Menschen mit einem hohen Risiko behandeln, betreuen oder pflegen (insbesondere in der Hämatoonkologie und Transplantationsmedizin)*	Mehr als 8 Mio.
**Hohe Priorität** Über 70-Jährige Personen mit Trisomie 21, mit Demenz oder geistiger Behinderung, nach einer Organtransplantation Enge Kontaktpersonen von solchen pflegebedürftigen Personen, die über 70 Jahre alt sind, an Trisomie 21 oder einer geistigen Behinderung (bzw. Demenz) leiden oder nach einer Organtransplantation ein hohes Infektionsrisiko haben Kontaktpersonen von Schwangeren Personen, die in stationären Einrichtungen für geistig behinderte Menschen tätig sind oder im Rahmen ambulanter Pflegedienste regelmäßig geistig behinderte Menschen behandeln, betreuen oder pflegen *Personen, die in Bereichen medizinischer Einrichtungen mit einem hohen oder erhöhten Expositionsrisiko in Bezug auf das Corona-Virus SARS-CoV‑2 tätig sind, insbesondere Ärzte und sonstiges Personal mit regelmäßigem Patientenkontakt, Personal der Blut- und Plasmaspendedienste und in SARS-CoV-2-Testzentren* Personen im öffentlichen Gesundheitsdienst und in relevanten Positionen der Krankenhausinfrastruktur	Mehr als 7 Mio.
**Erhöhte Priorität** Über 60-Jährige Personen mit folgenden Krankheiten: Adipositas, chronische Nierenerkrankung, chronische Lebererkrankung, Immundefizienz oder HIV-Infektion, Diabetes mellitus, div. Herzerkrankungen, Schlaganfall, Krebs, COPD oder Asthma, *Autoimmunerkrankungen und Rheuma* Beschäftigte in medizinischen Einrichtungen mit niedrigem Expositionsrisiko (Labore) und ohne Betreuung von Patienten mit Verdacht auf Infektionskrankheiten	Mehr als 5 Mio.

*kursiv:* relevante Passagen für Patienten mit rheumatologischen Erkrankungen und medizinischen Personal

*SARS-CoV-2* severe acute respiratory syndrome coronavirus 2, *COPD* chronic obstructive pulmonary disease

Aus dieser STIKO-Empfehlung hat das BGM die Reihenfolge der Impfungen in einer Rechtsverordnung festgelegt, die rückwirkend ab 15.12.2020 in Kraft getreten ist [9]. Diese Coronavirus-Impfverordnung – CoronaImpfV (ImpfVo) beschließt zunächst eine Priorisierung in 3 Gruppen (höchste-hohe-erhöhte Priorität) und sieht folgende Reihenfolge vor (Auszüge in Tab. 5 unten). In der Spalte 2 sind die geschätzten Personenzahlen gemäß STIKO angegeben.

Aus Sicht der DGRh ist das Vorgehen der STIKO-Empfehlung mit dem Konzept einer Priorisierung in Zeiten begrenzter Impfstoffressourcen alternativlos. Die Ergebnisse der STIKO-Analyse decken sich mit den Daten aus den laufenden COVID-19-Registern: Patienten mit entzündlich rheumatischen Erkrankungen haben in der Regel ein nur leicht erhöhtes Risiko für eine Hospitalisierung und/oder einen schweren Verlauf gegenüber der Normalbevölkerung. Die momentan noch unzureichende Datenlage zu COVID-19 macht aber eine exakte Risikoabschätzung für den einzelnen Patienten mit einer entzündlich rheumatischen Erkrankung schwierig. Einzelne (seltene) Erkrankungen oder Therapien sind in den COVID-19-Registern möglicherweise (noch) unterrepräsentiert. Auch werden in den Registern teilweise Erkrankungsstadien oder Schweregrade nicht eindeutig erfasst. Eine Analyse des kumulativen Risikos durch mehrere Risikofaktoren gibt es nicht.

Die DGRh würde sich – genau wie auch andere Fachgesellschaften für deren Patientengruppen mit entsprechend erhöhtem Risiko – deshalb wünschen, dass bei besserer Verfügbarkeit der Impfstoffe die Definition der Personengruppe mit entzündlichen rheumatischen Erkrankungen genauer differenziert wird und neben der Gruppe „erhöhte Priorität“ (Stufe 3) eine Gruppe von Patienten mit „hoher Priorität“ (Stufe 2) für eine SARS-CoV-2-Impfung vorgesehen wird (s. hierzu auch Tab. 4 mit unterschiedlichen Vorschlägen der DGRh, der aktuellen Empfehlung der STIKO und der Grundlage der ImpfVo). In der laufenden Diskussion wird sich die DGRh deshalb weiter bemühen, eine genauere Differenzierung von Patienten mit entzündlich rheumatischen Erkrankungen in zukünftigen Vorgaben der STIKO abzubilden. Damit sollen v. a. Patienten mit schwersten rheumatischen Erkrankungen oder in besonderen Therapiesituationen adäquat für die Impfung berücksichtigt werden. In der aktualisierten Empfehlung vom 08.01.2021 hält die STIKO fest, dass bei der Priorisierung innerhalb der Impfempfehlung nicht alle Krankheitsbilder oder Impfindikationen berücksichtigt werden können. Es sind deshalb auch Einzelfallentscheidungen möglich, und es obliegt den für die Impfung Verantwortlichen, nicht explizit genannte Personen in die jeweilige Priorisierungskategorie einzuordnen. Gemäß STIKO betrifft dies u. a. auch Personen mit seltenen, schweren Vorerkrankungen, für die bisher zwar keine ausreichende wissenschaftliche Evidenz bezüglich des Verlaufes einer COVID-19-Erkrankung vorliegt, für die aber ein erhöhtes Risiko angenommen werden kann. Somit bleibt Rheumatologen/-innen eine Möglichkeit, über entsprechende Atteste eine Höherpriorisierung für bestimmte Patienten zu erwirken. Einen Anhalt, für welche Patienten dies infrage kommt, kann Tab. 4 geben.

## Atteste

Für Bewohner von Alten- und Pflegeheimen und Patienten, die das 80. bzw. 70. Lebensjahr erreicht haben (Gruppe der mit „höchster“ und „hoher“ Priorität), sind keine Atteste auszustellen, da die Priorisierung allein über das Alter bzw. die Betreuungssituation erfolgt. Bei anderen Zugehörigen der höchsten und hohen Priorisierungsgruppe ist die Impfberechtigung über die berufliche Tätigkeit mittels einer Bescheinigung des Arbeitgebers nachzuweisen. Laut Impfverordnung benötigen jedoch Patienten mit Vorerkrankungen der Priorisierungsstufen 2 und 3 ein ärztliches Attest, damit sie ihren Anspruch auf eine vorrangige Impfung nachweisen können. Eine erhöhte Priorität für die Corona-Impfung alleine aufgrund einer chronischen Erkrankung ist mittels eines Attestes nachzuweisen. Beispiel für eine formlose Bescheinigung für Patienten mir rheumatologischen Erkrankungen kann sein: „Bei Frau K.Mustermann liegt eine Erkrankung im Sinne von Paragraf 4 Ziffer 2 der Impfverordnung vor (Priorisierungsgruppe 3).

Für die Ausstellung von in diesem Zusammenhang erforderlichen Attesten erhalten Ärzte entsprechend der KBV bzw. der Impfverordnung eine pauschale Vergütung von 5 € plus 0,90 € für Versand. Zur Abrechnung hat die KBV nach der Impfverordnung entsprechende Vorgaben rückwirkend zum 15.12.2020 beschlossen. Ärzte können ab diesem Datum Atteste ausstellen und abrechnen. Die entgültige Regelungen hat die Kassenärztliche Bundesvereinigung (KBV) gerade festegelegt (https://www.kbv.de/html/1150_50250.php).

## Medizinisches Personal

Nach der Impfverordnung sollen Personen mit Kontakten zu besonders vulnerablen Gruppen mit höchster Priorität geimpft werden (Tab. 5). Die STIKO empfiehlt explizit Personen mit engem Kontakt zu schwer immunsupprimierten/onkologischen/transplantierten Patient/-innen in der höchsten Priorisierungstufe 1 zu impfen. Die DGRh sieht dies für Rheumatologen/-innen als gegeben an.

Die Kassenärztliche Bundesvereinigung zählt zur zweiten Stufe (mit hoher Priorität) u. a. „Personen, die in Bereichen medizinischer Einrichtungen mit hohem oder erhöhtem Expositionsrisiko arbeiten, insbesondere Ärzte und sonstiges Personal mit regelmäßigem unmittelbaren Patientenkontakt“. Dagegen zählen „Personen, die in medizinischen Einrichtungen mit niedrigem Expositionsrisiko tätig sind, beispielsweise in Laboren, und Personal, das keine Patienten mit Verdacht auf Infektionskrankheiten betreut, zur Kategorie 3 mit erhöhter Priorität“. Es obliegt den leitenden Ärzten einer jeden medizinischen Einrichtung, eine Einteilung für das eigene Personal durchzuführen und entsprechende Bescheinigungen auszustellen (s. oben bei „Atteste“).

## Fazit

- Die Impfung rheumatologischer Patienten mit COVID-19-Impfstoffen ist möglich.
- Die Impfung rheumatologischer Patienten ist in der Regel zu empfehlen.
- Atteste sind nur bei unter 60-jährigen rheumatologischen Patienten notwendig.
- Die Details zur Umsetzung der Impfstrategie sind regional variabel.
